# Healthy lifestyle, metabolomics and incident type 2 diabetes in a population-based cohort from Spain

**DOI:** 10.1186/s12966-021-01219-3

**Published:** 2022-01-27

**Authors:** Mario Delgado-Velandia, Vannina Gonzalez-Marrachelli, Arce Domingo-Relloso, Marta Galvez-Fernandez, Maria Grau-Perez, Pablo Olmedo, Iñaki Galan, Fernando Rodriguez-Artalejo, Nuria Amigo, Laisa Briongos-Figuero, Josep Redon, Juan Carlos Martin-Escudero, Daniel Monleon-Salvado, Maria Tellez-Plaza, Mercedes Sotos-Prieto

**Affiliations:** 1 Department of Preventive Medicine and Public Health. School of Medicine, Universidad Autonoma de Madrid; Instituto de Investigacion Sanitaria Hospital Universitario La Paz (IdiPaz), Madrid, Spain; 2 Department of Physiology, School of Medicine, University of Valencia, Valencia, Spain; 3 Institute for Biomedical Research Hospital Clinic de Valencia INCLIVA, Valencia, Spain; 4 Integrative Epidemiology Group, Department of Chronic Diseases Epidemiology, National Center for Epidemiology, Carlos III Health Institutes, Madrid, Spain; 5 Department of Environmental Health Sciences, Columbia University Mailman School of Public Health, New York, NY USA; 6 Department of Statistics and Operations Research, University of Valencia, Valencia, Spain; 7 Department of Legal Medicine and Toxicology. School of Medicine, University of Granada, Granada, Spain; 8 Department of Chronic Diseases Epidemiology, National Center for Epidemiology, Carlos III Health Institute, Monforte de Lemos, 5, 28029 Madrid, Spain; 9 CIBERESP (CIBER of Epidemiology and Public Health), Madrid, Spain; 10 IMDEA-Food Institute, CEI UAM+CSIC, Madrid, Spain; 11 Biosfer Teslab, Av. Universitat, 1 43204 Reus, Spain; 12 Department of Basic Medical Sciences, University Rovira I Virgili, Reus, Spain; 13 CIBERDEM (CIBER of Diabetes and Metabolic Diseases), Madrid, Spain; 14 Department of Internal Medicine, University Hospital Rio Hortega, Valladolid, Spain; 15 CIBERFES (CIBER of Frailty and Healthy Aging), Madrid, Spain; 16 Department of Pathology, University of Valencia, Av. Blasco Ibañez, 15, 46010 Valencia, Spain; 17 Department of Environmental Health, Harvard T.H. Chan School of Public Health, Boston, MA USA

**Keywords:** Cohort study, Healthy lifestyle, Metabolomics, Spanish population, Type 2 diabetes

## Abstract

**Background:**

The contribution of metabolomic factors to the association of healthy lifestyle with type 2 diabetes risk is unknown. We assessed the association of a composite measure of lifestyle with plasma metabolite profiles and incident type 2 diabetes, and whether relevant metabolites can explain the prospective association between healthy lifestyle and incident type 2 diabetes.

**Methods:**

A Healthy Lifestyle Score (HLS) (5-point scale including diet, physical activity, smoking status, alcohol consumption and BMI) was estimated in 1016 Hortega Study participants, who had targeted plasma metabolomic determinations at baseline examination in 2001–2003, and were followed-up to 2015 to ascertain incident type 2 diabetes.

**Results:**

The HLS was cross-sectionally associated with 32 (out of 49) plasma metabolites (2.5% false discovery rate). In the subset of 830 participants without prevalent type 2 diabetes, the rate ratio (RR) and rate difference (RD) of incident type 2 diabetes (n cases = 51) per one-point increase in HLS was, respectively, 0.69 (95% CI, 0.51, 0.93), and − 8.23 (95% CI, − 16.34, − 0.13)/10,000 person-years. In single-metabolite models, most of the HLS-related metabolites were prospectively associated with incident type 2 diabetes. In probit Bayesian Kernel Machine Regression, these prospective associations were mostly driven by medium HDL particle concentration and phenylpropionate, followed by small LDL particle concentration, which jointly accounted for ~ 50% of the HLS-related decrease in incident type 2 diabetes.

**Conclusions:**

The HLS showed a strong inverse association with incident type 2 diabetes, which was largely explained by plasma metabolites measured years before the clinical diagnosis.

**Supplementary Information:**

The online version contains supplementary material available at 10.1186/s12966-021-01219-3.

## Background

Diabetes is a highly prevalent disease -at least 463 million people aged 20–79 years in 2019, and a major cause of disability and death [1]. The number of people with diabetes is expected to increase [1]. Type 2 diabetes has been associated with a number of risk factors that are both non-modifiable (age, genetics) and modifiable (environmental including lifestyle) [2]. Since genetic variation explained less than ~ 15% of the type 2 diabetes heritability, it is suspected that the environment and lifestyle have a more relevant role in type 2 diabetes development [2]. Diet, physical activity, body mass index (BMI), smoking and alcohol consumption have been individually associated with increased type 2 diabetes risk [3]. Previous studies jointly evaluating multiple healthy lifestyle behaviours found greater reductions in type 2 diabetes risk compared to the expected reduction from the individual lifestyle factors [4, 5].

Metabolomics —the determination of intermediary molecules and metabolism by-products [6]— offer opportunities to understand biological pathways that are potentially influenced by lifestyles and can help identifying strategies for type 2 diabetes precision prevention [7]. Several lifestyle factors have been associated with individual metabolic markers [8, 9]. Alternatively, individual metabolites have been associated with different stages in the type 2 diabetes progression [8, 10]. For instance, in a meta-analysis of 19 prospective studies, specific branched chain and aromatic amino acids were associated with both pre-diabetes and type 2 diabetes [10]. Furthermore, some of the metabolites associated to type 2 diabetes have also been related to specific lifestyle factors -glutamine for alcohol consumption [11], and branch-chain amino acids for physical activity [12] and obesity [13]. However, the contribution of metabolomic profiles to explain the association of a composite measure of overall lifestyle with type 2 diabetes risk is unknown.

Therefore, the aim of this study was to assess the association between adherence to a healthy lifestyle (measured by the Healthy Lifestyle Score [HLS]) with metabolic profiles and incident type 2 diabetes. In order to identify the most relevant metabolites in our data, we also used a probit extension of Bayesian Kernel Machine Regression (BKMR-P), which allowed to evaluate the prospective association of simultaneously-modelled metabolites with type 2 diabetes, as it can handle correlations and high-order interactions between metabolites mixtures [14, 15]. We subsequently evaluated whether HLS-related differences in relevant metabolites can explain the prospective association between healthy lifestyle and incident type 2 diabetes after a 14-year follow-up.

## Methods

### Study Participants

The Hortega Study is a population-based cohort representative of a general population from Valladolid, Spain [16]. Details of the study design and data collection methods have been described elsewhere [16]. The study population consisted of 1502 beneficiaries of the universal public health system assigned to the University Hospital Rio Hortega (UHRH) catchment area. Baseline physical examination (2001–2003) included validated questionnaires and laboratory assessment of standard biochemical profiles, and collection of plasma samples for metabolomics. In 2015, two physician reviewers blindly assessed the participants’ medical records and adjudicated health events that occurred during follow-up. The study protocol was approved by the institutional review board at UHRH and written informed consent was obtained from all participants [16].

Out of the 1502 recruited individuals, we sequentially excluded participants missing information on metabolomics (*n* = 299), smoking status (*n* = 2), educational level (*n* = 3), BMI *(n* = 40), diet (*n* = 141), and leisure time physical activity (*n* = 1) leaving 1016 participants for the cross-sectional analysis. The participant characteristics comparing excluded and included participants were similar (Supplementary Table S1, Additional File 1). We additionally excluded participants with prevalent type 2 diabetes at baseline (*n* = 94) and participants lost to follow-up (*n* = 92), leaving 830 participants for the prospective analyses of lifestyle- related metabolites and incident type 2 diabetes (See Supplementary Fig. S1, Additional File 1).

### Type 2 Diabetes assessment

Blood samples were collected after a mean fasting time of 3 h (range 0–17 h). Glycaemia was determined through the glucose oxidase method using a Hitachi 704 analyzer (Boehringer Mannheim, Germany). Participants with non-fasting glucose levels ≥7.8 mmol/l underwent second fasting glucose and glycosylated hemoglobin (HbA1c) determinations. HbA1c was measured from capillary blood samples using a DCA 2000 HbA1c analyzer (Bayer Diagnostics, Tarrytown, NY, USA). Participants were considered as prevalent type 2 diabetes cases if they had medical diagnosis before the baseline examination or there were records of diabetes medication use on their medical history; or if their baseline fasting plasma glucose was ≥7.0 mmol/l or HbA1c was ≥48 mmol/mol (≥6.5%). Participants were considered as incident type 2 diabetes cases if they were diabetes-free at baseline examination and the diagnosis of type 2 diabetes on their medical record met the diabetes definition during follow-up [16]. The validity of electronical medical records for the ascertainment of type 2 diabetes in the context of epidemiological studies within the Spanish universal public health system has been evaluated before [17]. In a subsample of public health system beneficiaries from Madrid [17], electronic health records showed adequate positive and negative predictive values (87.9 and 97.3%, respectively) for the identification of type 2 diabetes.

### Healthy Lifestyle Score

The Healthy Lifestyle Score (HLS) was estimated at baseline and included five-components (diet, physical activity, smoking status, alcohol consumption and BMI), following a well-established approach [18]. Scores for each component were 0 points (non-adherence) or 1 point (adherence) with a total range of 0–5 points, with a higher score indicating higher adherence to a healthy lifestyle. All women and men had daily energy intakes ranging 2092 to 14,644 kJ/day (500 to 3500 kcal/day), and 3347.2 and 16,736 kJ/day (800 to 4000 kcal/day), respectively. The alternate Mediterranean Diet score (aMED) measured the degree of adherence to a Mediterranean diet [19], instead of the Alternate Healthy Eating Index (AHEI) [18], since the aMED score is more appropriate to our study population. The aMED score was derived by excluding the alcohol consumption item because it was already a component of the HLS. As a result, the aMED score ranged 0–8 points. Healthy diet (1 point) was defined as an aMED score in the top 40% of the distribution (aMED ≥5). Women with alcohol intakes between 5 and 15 g/day and men with alcohol intakes between 5 and 30 g/day were given 1 point; all other participants received none [18]. Leisure-time physical activity was assessed as type of sports practiced and amount of time practicing each sport per week. The METs-minute/week (Metabolic Equivalent of Task-minute/week) were calculated using the equivalences in the Compendium of Physical Activities 2011 [20]. Participants received 1 point if they achieved at least 600 METs-minute/week performing moderate or vigorous leisure time physical activities, as recommended by the World Health Organization [21]. The BMI was derived from height and weight measured in standardized conditions; a BMI 18.5–24.9 kg/m^2^ was considered as healthy (1 point). Participants self-identified as never smokers were awarded 1 point; former and current smokers received none. Finally, we categorized the HLS in low (0–1 points), medium (2 points), and high adherence (3–5 points) groups.

### Metabolites Assessment

Metabolites levels were measured at baseline through nuclear magnetic resonance (NMR) using a Bruker Avance DRX 600 spectrometer (Bruker GmbH, Germany). The chemical shift region studied was between 0.50–4.70 ppm (ppm). The obtained spectra were normalized to total aliphatic spectral area after being binned into buckets of 0.01 ppm. The signals of the targeted metabolites were processed using in-house routines for MATLAB V.6.5. The results were confirmed through superposition of normalized serum spectra derived from two-dimensional NMR methods, namely homonuclear correlation spectroscopy and heteronuclear single quantum correlation spectroscopy. An extended lipoprotein profile was evaluated using the LIPOSCALE® method for NMR spectra [22, 23], and included lipoprotein lipid composition and size of the three main classes (VLDL, LDL and HDL) and the particle concentration of their respective subclasses (large, medium, and small). Particle concentrations and lipoprotein subtypes were determined using the distinctive signals of the lipid methyl group. Lipid concentration were converted to lipid volumes using common conversion factors [23]. The available set of metabolites to conduct the study objectives included 12 amino acids, 6 fatty acids, 5 products of bacterial co-metabolism, 17 lipoprotein subclasses, the sphingolipid-related O-phosphoethanolamine, 2 fluid balance and 6 energy metabolism-related metabolites. We adjusted all metabolites measures to the number of fasting hours at the time of plasma sample collection using linear regression and recalibrated the resulting residuals to the mean metabolite concentrations in the study population.

### Other variables

Information on education was self-reported. Prevalent dyslipidemia was defined as either lipid-lowering medication use recorded on medical history or as a non-fasting total cholesterol > 5.2 mmol/l. Prevalent hypertension was defined as systolic/diastolic blood pressure (average between two measurements with a 5-min interval assessed by trained personal) > 140/90 mmHg; or a medical record with prior hypertension diagnosis or blood pressure-lowering medication use.

### Statistical analysis

#### Descriptive analysis and association of HLS with metabolites

In order to account for the complex sampling design and survey weights, we conducted the statistical analyses using the “survey” package in R software (version 4.0.2, R Core Team 2020). We summarized the participants’ characteristics using descriptive statistics (mean and proportions). We descriptively estimated the survey-weighted type 2 diabetes incidence rate by using generalized linear models as conducted with the svyglm() command from the R survey package with family Poisson and link log, which included an offset term for the individual log-transformed person-years of follow-up and no covariates. For metabolic data, we calculated median and interquartile range by HLS categories. In non-exploratory analysis, the type I error probability threshold was generally set to 0.05 (two tailed). However, the cross-sectional evaluation of adherence to the HLS with individual metabolites in separate linear regression models, was exploratory. In order to account for multiple exploratory testing in this context, we set a false discovery rate (FDR) significance threshold of 2.5% as conducted by the R package “qvalue” [24], with a lambda parameter set to 0. We conducted two progressively adjusted models: Model 1 adjusted for age (years), sex (male, female) and education (≤ high school, > high school); and Model 2 included Model 1 and prevalent hypertension (no, yes), total plasma cholesterol (mg/dL) and use of lipid-lowering medication (no, yes).

#### Association of HLS and incident type 2 diabetes

We estimated adjusted rate ratios (RR) and rate differences (RD) per 10,000 person-years of incident type 2 diabetes, by adherence to the baseline HLS (categorized and continuous) using Poisson and Aalen additive hazards models, respectively. Given the controversial evidence on the protective effect of alcohol consumption on type 2 diabetes risk [25], we conducted sensitivity analysis: a) including alcohol in both aMED [19] and HLS scores definition; b) excluding alcohol from both aMED and HLS scores definition; c) with non-drinkers being also awarded 1 point in the alcohol consumption component of HLS. In secondary analyses, we examined the associations of HLS and type 2 diabetes by subgroups defined by sex, education, and prevalent dyslipidemia and hypertension status introducing interaction terms in the regression models.

#### Association of metabolites with incident type 2 diabetes

First, we estimated fully adjusted rate ratios (RR) and rate differences (RD) of incident type 2 diabetes by individual HLS-related metabolites using Poisson and Aalen additive hazard models, respectively. We re-scaled the resulting coefficients and confidence intervals to compare the 90th to the 10th percentiles of each metabolite distribution in order to improve their interpretability. Second, we used BKMR to simultaneously evaluate the association of these metabolites with incident type 2 diabetes [15]. BKMR uses a flexible kernel to handle high dimensional correlations, to account for non-linearity and to provide an estimation of both individual and joint effect of compounds mixtures [15]. The R package BKMR conducts Bayesian inference for the probit regression model (BKMR-P), which we adapted to time-to-event survey data using a data augmentation approach [14]. The posterior inclusion probabilities (PIP) (from 0 to 1) obtained from the BKMR-P quantify how much the data favors the inclusion of a metabolite in the model.

Subsequently, to evaluate whether relevant metabolites contribute to explain the association of HLS and type 2 diabetes, we estimated the amount of avoided incident type 2 diabetes cases per 1-point HLS increase (per 10,000 person-years) that can be attributed to differences in metabolites levels, estimated as the relative change in the beta coefficient associated to HLS from the Aalen additive hazard models when each metabolite group was introduced in the model (i.e. the relative amount of association explained by metabolites was estimated as [1 – (difference in type 2 diabetes rates per one HLS point increase in models adjusting for specific metabolites group / difference in type 2 diabetes rates per one HLS point increase in the reference model without metabolites)] × 100). Additive hazard models are recommended to study the contribution of intermediate variables in survival settings [26].

In confirmatory post-hoc analysis, we used formal causal mediation analysis for survival outcomes [26, 27], to evaluate whether the sum of estimated relative mediated effects for the most relevant individual metabolites did equal the percent explained in the association of HLS and incident diabetes with and without relevant metabolites entered as a group (as expected when the causal mediation assumptions hold, and the individual metabolites are not causally correlated). In particular we used the product of coefficients method to calculate natural indirect effects. The Aalen additive hazards outcome model included time to incident diabetes as the outcome, HLS as the exposure and most relevant metabolites (i.e., those identified by the BKMR analysis) as mediators. The mediator models were linear models where each relevant metabolite was entered as the dependent variable in separate mediator models and HLS (exposure) was entered as the independent variable. Both outcome and mediator models were adjusted for age, sex, education, prevalent hypertension, total plasma cholesterol, use of lipid-lowering medication and the other relevant metabolites. As result, absolute mediated effects (natural indirect effects) were also reported as the number of avoided incident type 2 diabetes cases per 1 HLS-point increase (per 10,000 person-years) that can be independently attributed to differences in specific metabolites levels after accounting for other relevant metabolites. The relative mediated effect was calculated as the ratio between mediated effects and adjusted changes in diabetes cases per 1 HLS-point increase before adding the specific metabolite to the model. Confidence intervals were calculated using a resampling method that takes random values from multivariate normal distribution of the estimates [26, 27].

## Results

### Descriptive analysis

In our study population the mean age was 48.5 years and 49% of participants were males (Table 1). Participants with higher adherence to the HLS were more likely to be younger and female, with lower prevalence of dyslipidemia and hypertension (Table 1). The median HLS was 2 points. Never smoking was the HLS component for which the participants had the highest compliance with the recommendations, while alcohol consumption had the lowest (See Supplementary Table S2, Additional File 1). Lipoprotein composition and particle concentrations including VLDL, LDL and IDL cholesterol and triglycerides; large, medium and small VLDL, and small LDL, as well as, −CH_2_N fatty acids and acetone concentrations progressively decreased across the HLS categories (See Supplementary Table S3, Additional File 1). Increasing HLS categories showed increasing concentrations of other metabolites such as amino acids, citrate, pyruvate, 3-hydroxybutyrate, isopropanol, trimethylamines or phenylpropionate (See Supplementary Table S3, Additional File 1). Participants with incident type 2 diabetes were more likely to be older, with lower educational level and higher prevalence of dyslipidemia and hypertension (See Supplementary Table S4, Additional File 1).

**Table 1:** Participants characteristics by Healthy Lifestyle Score categories in the Hortega Study (*n* = 1016)

	Overall	Low HLS (0–1 points)	Moderate HLS (2 points)	High HLS (3–5 points)
*n* (%)	1016	491 (46.41)	310 (30.39)	215 (23.20)
Age, years, mean (SE)	48.54 (17.07)	51.41 (16.96)	49.88 (16.67)	41.03 (15.54)
Sex, Male, *n* (%)	511 (48.96)	280 (55.50)	126 (39.87)	105 (47.80)
Education, ≤High school, *n* (%)	725 (68.99)	374 (74.65)	227 (70.55)	124 (55.63)
Energy intake, Kilocalories/day, mean (SE)	2235.80 (705.81)	2193.93 (705.76)	2236.69 (697.55)	2318.41 (709.44)
Prevalent type 2 diabetes, Yes, *n* (%)	94 (6.99)	59 (8.98)	30 (7.82)	5 (1.94)
Prevalent dyslipidaemia, Yes, *n* (%)	538 (51.74)	291 (58.94)	167 (52.92)	80 (35.81)
Prevalent hypertension, Yes, *n* (%)	441 (36.50)	259 (44.42)	131 (37.11)	51 (19.83)
Healthy Lifestyle Score (HLS) components				
BMI, kg/m^2^, mean (SE)	26.17 (4.18)	27.44 (4.01)	26.11 (4.41)	23.72 (2.92)
Leisure time physical activity, METs-minute/week, mean (SE)	463.57 (1280.05)	149.13 (607.17)	324.36 (770.99)	1274.99 (2160.09)
8-point aMED (alcohol excluded), mean (SE)	3.70 (1.62)	3.11 (1.30)	4.06 (1.68)	4.42 (1.70)
Alcohol intake, grams/day, mean (SE)	11.81 (21.97)	15.21 (28.80)	7.93 (13.14)	10.08 (12.20)
Smoking status, Never smoker, *n* (%)	466 (43.64)	137 (23.69)	179 (54.92)	150 (68.76)

Abbreviations: *BMI* Body Mass Index; *aMED* alternate Mediterranean Diet score; *HLS* Healthy Lifestyle Score; *MET* Metabolic Equivalent of Task; *SE* standard error

### Cross-sectional association of HLS and metabolites

At a FDR of 2.5% HLS was associated with 32 out of 49 plasma metabolites (Table 2). The most frequently associated metabolites were the lipoprotein particles subclasses and content group (*n* = 10) (mean difference [MD] [95% CI] ranged from − 19.43 [− 29.00, − 9.87] nmol/l for small LDL to − 0.005 [− 0.01, − 0.002] mmol/l for IDL triglycerides); followed by the amino acids group (*n* = 8) (MD [95%CI] ranged from 0.11 [0.06, 0.17] for alanine, to 0.01 [0.004, 0.02] for cysteine); the energy metabolism group (*n* = 5) (MD ranged from − 0.10 [− 0.16, − 0.05] for acetone; to 0.02 [0.01, 0.03] for pyruvate); products of bacterial co-metabolism (*n* = 3) (MD were 0.09 [0.05, 0.14] for phenylpropionate; to 0.05 [0.03, 0.08] for isopropanol); and fluid balance (*n* = 2) (MD were 0.07 [0.03, 0.11] albumin; and 0.02 [0.01, 0.03] for creatinine).

**Table 2:** Mean difference (95%CI) of standardized NMR-metabolites per 1-point increase in HLS in the Hortega Study^a^

		Model 1		Model 2	
Group	Metabolite	MD (95% CI)	*p*-value	MD (95% CI)	*p*-value
Lipoprotein profile	*Cholesterol, mmol/l*				
	VLDL cholesterol	−0.07 (− 0.09, − 0.04)	< 0.001	− 0.05 (− 0.07, − 0.03)	< 0.001*
	LDL cholesterol	−0.11 (− 0.17, − 0.05)	< 0.001	−0.03 (− 0.06, 0.002)	0.04
	HDL cholesterol	0.04 (0.02, 0.07)	0.001	0.05 (0.02, 0.07)	< 0.001*
	IDL cholesterol	−0.02 (− 0.03, − 0.01)	< 0.001	−0.01 (− 0.02, − 0.003)	0.006*
	*Triglycerides, mmol/l*				
	VLDL triglycerides	−0.13 (− 0.19, − 0.08)	< 0.001	−0.1 (− 0.14, − 0.05)	< 0.001*
	LDL triglycerides	−0.007 (− 0.01, − 0.002)	0.006	−0.001 (− 0.005, 0.002)	0.43
	HDL triglycerides	−0.003 (− 0.01, 0.0007)	0.11	−0.002 (− 0.01, 0.002)	0.24
	IDL triglycerides	−0.007 (− 0.01, − 0.04)	< 0.001	−0.005 (− 0.01, − 0.002)	0.003*
	*Lipoprotein particle concentration*				
	Large VLDL, *nmol/L*	−0.18 (− 0.27, − 0.09)	< 0.001	−0.13 (− 0.21, − 0.06)	< 0.001*
	Medium VLDL, *nmol/L*	−1.13 (− 1.64, − 0.62)	< 0.001	−0.78 (− 1.24, − 0.33)	< 0.001*
	Small VLDL, *nmol/L*	− 7.34 (− 10.21, − 4.47)	< 0.001	−5.33 (− 7.85, − 2.80)	< 0.001*
	Large LDL, *nmol/L*	−4.58 (− 7.54, − 1.63)	0.002	− 0.98 (− 2.79, 0.83)	0.29
	Medium LDL, nmol/L	− 10.11 (− 19.62, − 0.6)	0.04	1.19 (− 4.59, 6.97)	0.69
	Small LDL, nmol/L	−35.75 (− 50.87, − 20.63)	< 0.001	−19.43 (− 29.00, − 9.87)	< 0.001*
	Large HDL, μmol*/L*	0.001 (− 0.003, 0.01)	0.68	0.003 (0.0002, 0.01)	0.04
	Medium HDL, *μmol/L*	0.22 (0.06, 0.38)	0.008	0.24 (0.08, 0.4)	0.003*
	Small HDL *μmol/L*	0.25 (− 0.08, 0.57)	0.13	0.35 (0.02, 0.67)	0.04
Amino acids ^b^	Alanine	0.14 (0.08, 0.20)	< 0.001	0.11 (0.06, 0.17)	< 0.001*
	Creatine phosphate	0.02 (0.01, 0.03)	< 0.001	0.01 (0.002, 0.02)	0.01*
	Creatine	0.02 (0.01, 0.03)	< 0.001	0.01 (0.005, 0.02)	< 0.001*
	Cysteine	0.02 (0.01, 0.02)	< 0.001	0.01 (0.004, 0.02)	0.002*
	Glutamine	0.11 (0.07, 0.15)	< 0.001	0.08 (0.04, 0.12)	< 0.001*
	N-acetylglutamine	0.08 (0.05, 0.11)	< 0.001	0.06 (0.03, 0.08)	< 0.001*
	Proline	0.12 (0.07, 0.16)	< 0.001	0.09 (0.05, 0.12)	< 0.001*
	Tryptophan	0.03 (0.01, 0.06)	0.02	0.02 (0, 0.05)	0.06
	Tyrosine	0.02 (0.01, 0.04)	0.002	0.01 (0, 0.02)	0.08
	Isoleucine	0.05 (0.02, 0.08)	0.003	0.04 (0, 0.07)	0.02
	Leucine	0.05 (0.02, 0.08)	< 0.001	0.04 (0.01, 0.07)	0.01*
	Valine	0.05 (0.02, 0.09)	0.003	0.04 (0.01, 0.08)	0.02
Fatty acids ^b^	CH_2_CH_2_CO	−0.19 (−0.29, −0.09)	< 0.001	−0.14 (− 0.24, − 0.04)	0.006*
	CH_2_CH_3_	− 0.13 (− 0.24, − 0.03)	0.009	− 0.07 (− 0.17, 0.03)	0.15
	CH_2_N	−3.17 (−4.44, −1.89)	< 0.001	−2.17 (−3.33, −1.00)	< 0.001*
	CH_3_	−0.38 (− 0.61, − 0.15)	0.001	−0.19 (− 0.37, − 0.003)	0.05
	CHCH_2_CH	− 0.03 (− 0.08, 0.03)	0.33	−0.003 (− 0.05, 0.05)	0.92
	Isobutyrate	0.04 (0.01, 0.07)	0.003	0.03 (0.01, 0.06)	0.01*
Fluid balance ^b^	Albumin	0.11 (0.07, 0.16)	< 0.001	0.07 (0.03, 0.11)	< 0.001*
	Creatinine	0.03 (0.01, 0.04)	< 0.001	0.02 (0.01, 0.03)	< 0.001*
Energy metabolism ^b^	*Glycolysis*				
	Citrate	0.07 (0.04, 0.09)	< 0.001	0.05 (0.03, 0.07)	< 0.001*
	Lactate	−0.34 (−0.6, − 0.09)	0.009	− 0.24 (− 0.48, 0.01)	0.06
	Pyruvate	0.03 (0.02, 0.04)	< 0.001	0.02 (0.01, 0.03)	< 0.001*
	*Ketone bodies*				
	Acetate	0.05 (0.03, 0.07)	< 0.001	0.03 (0.01, 0.05)	0.001*
	Acetone	−0.13 (− 0.19, − 0.08)	< 0.001	−0.10 (− 0.16, − 0.05)	< 0.001*
	3-Hydroxybutyrate	0.11 (0.07, 0.15)	< 0.001	0.08 (0.04, 0.12)	< 0.001*
Products of bacterial co-metabolism ^b^	Ethanol	−0.05 (− 0.16, 0.06)	0.39	−0.06 (− 0.16, 0.04)	0.24
	Isopropanol	0.08 (0.05, 0.11)	< 0.001	0.05 (0.03, 0.08)	< 0.001*
	Methanol	0.01 (0.002, 0.02)	0.008	0.01 (−0.0001, 0.01)	0.05
	Trimethylamines	0.09 (0.05, 0.12)	< 0.001	0.06 (0.03, 0.09)	< 0.001*
	Phenylpropionate	0.11 (0.07, 0.16)	< 0.001	0.09 (0.05, 0.14)	< 0.001*
Phosphoethanolamines ^b^	O-phosphoethanolamine	0.07 (0.04, 0.10)	< 0.001	0.04 (0.02, 0.07)	0.002*

Abbreviations: *MD* mean differences; *CI* confidence interval; *HLS* Healthy Lifestyle Score. ^a^ *n* = 1016

Model 1 was adjusted for age (years), sex (men, women) and education (≤ high school, > high school). Model 2 was model 1 additionally adjusted for prevalent hypertension (no, yes), total plasma cholesterol (mg/dL) and use of lipid lowering medication (no, yes)

^b^ We normalized the spectral vector to the total spectral area excluding residual water signals to minimize the effects of variable dilution of the sample. The metabolic content is therefore expressed in relative metabolic content (unitless)

* Significant at a 2.5% False Discovery Rate

### Prospective association of HLS and type 2 diabetes

The number of incident type 2 diabetes cases after a median follow-up time of 13.3 years was 51 (the survey-weighted incidence rate during the study period was 40.2 per 10,000 person-years). The fully adjusted RR of diabetes comparing the medium and high (2 and 3–5 points, respectively) to the low (0–1 points) HLS adherence categories were 0.83 (95%CI 0.44, 1.56) and 0.20 (95%CI 0.04, 0.88), respectively (See Supplementary Table S5, Additional File 1); and their corresponding differences in incident rates were − 7.15 (95%CI -34.31, 20.01) and − 24.47 (95%CI -43.87, − 5.06) (See Supplementary Table S6, Additional File 1). The corresponding estimates per 1 HLS point increase was 0.69 (95%CI 0.51, 0.93) for RR (See Supplementary Table S5, Additional File 1) and − 8.23 (95%CI -16.34, − 0.13) for RD (See Supplementary Table S6, Additional File 1). In sensitivity analysis, similar results were obtained when alcohol consumption was included in both aMED and HLS, when alcohol consumption was excluded from both aMED and HLS, and when non-drinkers were awarded 1 point in the alcohol consumption component of the HLS (See Supplementary Table S7, Additional File 1). We did not observe differential associations by subgroups (See Supplementary Table S8, Additional File 1).

### Association of metabolites and type 2 diabetes

All the HLS-related metabolites (together representing 7 metabolites’ groups), except IDL cholesterol and triglycerides and creatinine, were individually associated with incident type 2 diabetes. In Aalen additive hazard models, the greatest differences in incident type 2 diabetes rates (95%CI) comparing the 90th to the 10th percentiles of metabolites distributions were observed for small LDL in the lipoprotein particles subclasses and contents group (78.19 [16.82, 139.56]); for N-acetylglutamine in the amino acids group (− 71.46 [− 109.9, − 33.01]); for acetone in the energy metabolism group (72.26 [29.49, 115.03]); for trimethylamines in the products of bacterial co-metabolism group (− 68.58 [− 112.14, − 25.01]); and for albumin in the fluid balance group (− 70.49 [− 116.19, − 24.78]) (Table 3). In Poisson regression models, the association of HLS-related metabolites with incident type 2 diabetes was statistically significant and directionally consistent compared to results from Aalen regression models (Table 3). In BKMR analysis, the overall metabolites mixture was significantly and inversely associated with the type 2 diabetes risk (See Supplementary Fig. S2, Additional File 1). Phenylpropionate and medium HDL particles, which consistently showed and inverse association with incident type 2 diabetes (See Supplementary Fig. S3, Additional File 1), followed by small LDL particles, which consistently showed a positive association with incident type 2 diabetes (See Supplementary Fig. S3, Additional File 1), displayed posterior inclusion probabilities (PIPs) higher than 20% (PIPs were 1, 1 and 0.23 respectively) (See Supplementary Table S9, Additional File 1).

**Table 3:** Rate Ratio and Rate Difference per 10,000 person-years, for incident type 2 diabetes (95%CI) comparing the 90th to the 10th percentiles of HLS-related metabolites distributions^a^

		RR (95% CI)				RD (95%CI)			
Group	Metabolite	Model 1	*p*-value	Model 2	*p*-value	Model 1	*p*-value	Model 2	*p*-value
Lipoprotein profile	*Cholesterol*								
	VLDL cholesterol	2.12 (1.45–3.08)	< 0.001	2.09 (1.35–3.24)	< 0.001	59.62 (18.18, 101.06)	0.005	60.66 (16.58, 104.74)	0.007
	HDL cholesterol	0.21 (0.07–0.66)	0.008	0.28 (0.10–0.79)	0.02	−52.40 (−88.35, −16.45)	0.004	− 47.56 (− 83.74, − 11.37)	0.01
	IDL cholesterol	1.27 (0.75–2.15)	0.38	1.11 (0.61–2.01)	0.74	9.82 (− 15.46, 35.1)	0.45	6.12 (−24.57, 36.81)	0.70
	*Triglycerides*								
	VLDL triglycerides	2.07 (1.48–2.91)	< 0.001	2.02 (1.38–2.95)	< 0.001	58.25 (17.54, 98.95)	0.005	56.59 (14.06, 99.12)	0.009
	IDL triglycerides	1.25 (0.69–2.29)	0.46	1.14 (0.60–2.17)	0.69	9.79 (−18.15, 37.73)	0.49	8.52 (−22.84, 39.89)	0.59
	*Lipoprotein particle concentration*								
	Large VLDL	1.77 (1.36–2.31)	< 0.001	1.67 (1.25–2.24)	< 0.001	48.30 (8.03, 88.58)	0.02	45.59 (4.49, 86.69)	0.03
	Medium VLDL	1.56 (1.20–2.03)	< 0.001	1.48 (1.12–1.95)	< 0.001	43.70 (8.93, 78.48)	0.01	39.86 (3.85, 75.87)	0.03
	Small VLDL	2.34 (1.62–3.37)	< 0.001	2.40 (1.57–3.68)	< 0.001	61.85 (20.86, 102.85)	0.003	62.69 (19.54, 105.84)	0.004
	Small LDL	1.70 (1.20–2.40)	0.003	2.71 (1.52–4.82)	< 0.001	45.69 (6.59, 84.78)	0.02	78.19 (16.82, 139.56)	0.01
	Medium HDL	0.15 (0.05–0.49)	0.002	0.22 (0.07–0.66)	0.007	−54.12 (−84.41, −23.83)	< 0.001	−46.41 (−76.50, −16.31)	0.003
Amino acids	Alanine	0.33 (0.20–0.53)	< 0.001	0.38 (0.24–0.60)	< 0.001	−66.25 (− 106.80, − 25.70)	0.001	−60.72 (− 100.87, − 20.58)	0.003
	Creatine phosphate	0.43 (0.22–0.84)	0.01	0.36 (0.18–0.76)	0.007	−36.60 (− 70.68, − 2.52)	0.04	−51.06 (−95.96, − 6.15)	0.03
	Creatine	0.34 (0.20–0.59)	< 0.001	0.27 (0.14–0.51)	< 0.001	−49.69 (− 82.80, − 16.57)	0.003	−68.15 (−113.77, − 22.52)	0.003
	Cysteine	0.35 (0.20–0.60)	< 0.001	0.29 (0.15–0.55)	< 0.001	−43.89 (− 74.10, − 13.69)	0.004	−57.78 (−97.34, − 18.21)	0.004
	Glutamine	0.32 (0.18–0.57)	< 0.001	0.29 (0.15–0.53)	< 0.001	− 51.97 (−88.04, −15.91)	0.005	−62.53 (− 106.14, − 18.91)	0.005
	N-acetylglutamine	0.28 (0.18–0.44)	< 0.001	0.27 (0.16–0.44)	< 0.001	− 68.69 (−104.62, − 32.76)	< 0.001	−71.46 (−109.90, − 33.01)	< 0.001
	Proline	0.34 (0.19–0.62)	< 0.001	0.33 (0.18–0.61)	< 0.001	− 51.58 (− 88.78, −14.37)	0.007	−58.68 (− 100.91, − 16.44)	0.006
	Leucine	0.32 (0.20–0.51)	< 0.001	0.37 (0.23–0.59)	< 0.001	−68.75 (−107.06, − 30.45)	< 0.001	−60.81 (− 97.75, − 23.87)	0.001
Fatty acids	CH2CH2CO-	3.38 (2.07–5.52)	< 0.001	3.03 (1.87–4.91)	< 0.001	64.57 (27.25, 101.89)	< 0.001	60.88 (24.37, 97.39)	0.001
	CH2N-	2.79 (1.69–4.60)	< 0.001	2.77 (1.62–4.73)	< 0.001	53.28 (17.86, 88.69)	0.003	59.06 (18.42, 99.70)	0.004
	Isobutyrate	0.22 (0.13–0.37)	< 0.001	0.27 (0.16–0.46)	< 0.001	−73.15 (− 109.93, − 36.38)	< 0.001	−65.92 (−101.30, − 30.54)	< 0.001
Fluid balance	Albumin	0.34 (0.20–0.58)	< 0.001	0.26 (0.14–0.50)	< 0.001	−50.89 (− 84.27, −17.52)	0.003	−70.49 (− 116.19, − 24.78)	0.003
	Creatinine	0.65 (0.34–1.26)	0.21	0.56 (0.26–1.17)	0.12	− 16.96 (− 46.61, 12.68)	0.26	−22.07 (− 56.62, 12.49)	0.21
Energy metabolism	*Glycolysis*								
	Citrate	0.30 (0.16–0.57)	< 0.001	0.27 (0.14–0.55)	< 0.001	−49.90 (− 84.40, − 15.40)	0.005	− 58.56 (−98.87, − 18.26)	0.004
	Pyruvate	0.32 (0.18–0.59)	< 0.001	0.31 (0.16–0.58)	< 0.001	− 49.48 (− 84.04, − 14.92)	0.005	− 56.04 (−94.75, − 17.32)	0.005
	*Ketone bodies*								
	Acetate	0.28 (0.15–0.52)	< 0.001	0.27 (0.15–0.51)	< 0.001	− 62.62 (− 101.68, − 23.55)	0.002	− 68.92 (− 111.80, − 26.04)	0.002
	Acetone	3.19 (1.97–5.15)	< 0.001	2.88 (1.84–4.51)	< 0.001	75.63 (32.20, 119.05)	< 0.001	72.26 (29.49, 115.03)	< 0.001
	3-Hydroxybutyrate	0.36 (0.20–0.64)	< 0.001	0.34 (0.18–0.63)	< 0.001	− 48.61 (− 84.22, − 13.00)	0.007	− 56.27 (− 97.80, − 14.74)	0.008
Products of bacterial co-metabolism	Isopropanol	0.47 (0.23–0.93)	0.03	0.39 (0.18–0.85)	0.02	−31.46 (− 63.24, 0.31)	0.05	−43.23 (− 83.80, − 2.66)	0.04
	Trimethylamines	0.29 (0.16–0.53)	< 0.001	0.23 (0.12–0.45)	< 0.001	−52.59 (− 86.57, − 18.61)	0.002	−68.58 (− 112.14, − 25.01)	0.002
	Phenylpropionate	0.34 (0.21–0.54)	< 0.001	0.39 (0.26–0.59)	< 0.001	− 70.33 (− 112.40, − 28.25)	0.001	−66.12 (− 108.06, − 24.19)	0.002
Phosphoethanolamines	O-phosphoethanolamine	0.38 (0.20–0.72)	0.003	0.33 (0.17–0.65)	0.002	− 42.53 (− 77.86, −7.19)	0.02	−56.54 (− 101.38, − 11.69)	0.01

Abbreviations: *RR* rate ratio; *RD* rate difference; *CI* confidence interval. ^a^ *n* = 830

Model 1 was adjusted for age (years), sex (men, women) and education (≤ high school, > high school). Model 2 was model 1 additionally adjusted for prevalent hypertension (no, yes), total plasma cholesterol (mg/dL) and use of lipid lowering medication (no, yes)

### Contribution of metabolites to HLS-related type 2 diabetes

In models adjusting for age, sex, hypertension status, total cholesterol and lipid-lowering medication, 1-point increase in HLS was associated with 8.23 avoided incident diabetes cases/10,000 person-years (95% CI, 16.34, 0.13) after a 14-year follow-up (Table 4). This decrease in type 2 diabetes incidence rates (RD) was substantially attenuated when HLS and diabetes-related metabolites were sequentially introduced by metabolite groups in the adjusted Aalen model. Metabolites from the lipoprotein profile caused the greatest attenuation in estimated number of avoided type 2 diabetes incidence cases with a 45.9% change in the HLS coefficient [RD changed from − 8.23 (95% CI, − 16.34, − 0.13) to − 4.45 (− 12.65, 3.75) after lipoproteins subclasses adjustment], followed by amino acids, bacterial co-metabolism, energy, fluid balance, phosphoethanolamines and fatty acids metabolites (corresponding % change in the HLS-regression coefficient was 45.4, 38.0, 36.8, 25.6, 24.2, and 22.5 respectively) (Table 4). When most relevant metabolites (i.e. metabolites with a PIP greater than 20% in the BKMR analysis) were simultaneously introduced in the model, the corresponding attenuation in the HLS-regression coefficient was 52.5% (Table 4). In other words, differences in relevant plasma metabolite measured at baseline approximately explained ~ 4 out of the 8 avoided incident diabetes cases/10,000 person-years attributable to a 1-point increase HLS, adjusting for age, sex, educational level, total cholesterol and lipid-lowering medication. Results from confirmatory post-hoc causal mediation analysis were supportive of the analysis that evaluated the change in the HLS-diabetes association with and without relevant metabolites entered as a group because the sum of the relative mediated effects of pheylpropionate, medium HDL and small LDL particle concentrations from the product of coefficient method (from Supplementary Table S10) was essentially similar to the originally estimated percent of the HLS-diabetes association explained by the 3 metabolites simultaneously entered as a group (Table 4).

**Table 4:** Difference in type 2 diabetes incidence per 10,000 person-years per 1-point HLS increase^a^

Adjustment models	Difference in type 2 diabetes rates per 10,000 person-years (95% CI)	Percent explained, % (95% CI)^c^
Age, sex, education, prevalent hypertension, total plasma cholesterol, use of lipid lowering medication (Reference Model)	−8.23 (− 16.3, − 0.13)	Reference
Reference model + lipoprotein subclasses	−4.45 (− 12.65, 3.75)	45.9 (3.6, 238.7)
Reference model + amino acids	−4.49 (− 13.06, 4.08)	45.4 (7.2, 274.0)
Reference model + fatty acids	−6.38 (−15.00, 2.24)	22.5 (−11.2, 164.1)
Reference model + fluid balance	−6.12 (− 14.58, 2.34)	25.6 (4.4, 223.7)
Reference model + energy	−5.20 (−13.93, 3.54)	36.8 (0.3, 285.2)
Reference model + products of bacterial co-metabolism	−5.10 (− 13.82, 3.62)	38.0 (2.8, 266.2)
Reference model + O-phosphoethanolamine	−6.24 (− 14.79, 2.31)	24.2 (2.9, 211.3)
Reference model + phenylpropionate, and medium HDL and small LDL particle concentrations ^b^	−3.91 (− 12.25, 4.44)	52.5 (15.9, 384.3)

Abbreviations: CI, confidence interval

^a^ Aalen additive models with progressive degrees of adjustment; *n* = 830, 51 incident type 2 diabetes cases and 779 non-cases

^b^ Relevant metabolites defined as showing a PIP > 20% in BKMR-P regression

^*c*^ Bias-corrected and accelerated 95% confidence intervals from bootstrap based on 1000 resamplings as conducted by the *boot* R package

## Discussion

In this population-based cohort with a 14-year follow-up, the HLS, a composite healthy-lifestyle measure, was cross-sectionally associated to plasma metabolomic profiles mostly representing lipoprotein subclasses, amino acids, energy metabolism, fatty acids, products of bacterial co-metabolism and fluid balance metabolites. While most of these metabolites were individually associated with type 2 diabetes risk in single-metabolite models, phenylpropionate and medium HDL followed by small LDL particle concentrations largely drove the prospective association of jointly modelled metabolites with diabetes, and explained ~ 50% of avoided type 2 diabetes cases attributable to healthy lifestyle. Our results, thus, support that early metabolic changes related to lifestyle may have an impactful role in type 2 diabetes prevention.

The association of lifestyle and type 2 diabetes is widely known. The available evidence is based on several prospective studies of healthy lifestyle scores and incident type 2 diabetes [28, 29]; a meta-analysis of 14 prospective studies that evaluated the association between combined lifestyle factors and incident type 2 diabetes [3]; and a meta-analysis of randomized clinical trials that summarized the long-term effect of different combined lifestyle interventions in individuals at high risk of type 2 diabetes [30]. However, the contribution of plasma metabolites to explain the association of overall lifestyle and incident type 2 diabetes had not been evaluated before.

We observed a strong association of HLS with metabolites profiles reflecting several metabolic pathways. Scarce studies have previously evaluated the association between lifestyle —as a composite measure— with metabolomics measures. In the EPIC cohort, a modified healthy lifestyle index (diet, BMI, physical activity, lifetime alcohol, smoking, diabetes and hepatitis) was related to a serum metabolic signature composed of hexoses, glutamic acid, sphingomyelins and a phosphatidylcholine [8]. In our study, metabolites involved in related metabolic pathways, including several amino acids, as well as markers of energy metabolism, were consistently associated to healthy lifestyle adherence. Additionally, we identified other metabolites types, mainly lipoprotein subclasses; but also products from bacterial co-metabolism; fluid balance; fatty acids and O-phosphoethanolamine, which had not been previously investigated in relation to overall lifestyle.

Importantly, most of the identified HLS-related metabolites in our study were also prospectively associated with type 2 diabetes. Some components of the lipoprotein profile that were found to be positively associated to type 2 diabetes in our study, have been previously reported [specifically VLDL cholesterol [31], HDL cholesterol [10, 31, 32], VLDL triglycerides [31]; large and small VLDL [10] and small LDL [32]]. Similarly, components of the amino acids (e.g. alanine, creatine, glutamine, proline) [33] and the bacterial co-metabolism (phenylpropionate) [34, 35] groups, have been consistently associated to type 2 diabetes risk in other studies. However, the mechanisms that explain the associations between most of plasma metabolites measured in our study and type 2 diabetes remain largely unclear.

Evidence obtained from animal models shows that the exogenous administration of glutamine improves glucose tolerance [36], while the administration of creatine [37] or cysteine [38] decreases glycemia. Interestingly, this is the first study to find an association between plasma O-phosphoethanolamine levels with incident type 2 diabetes. In In-vitro studies, O-phosphoethanolamine up-stream precursor Sphingosine-1-Phosphate, counterbalanced insulin-resistance in peripheral tissues such as liver and muscle, and protected pancreatic beta cells from apoptosis [39]. Moreover, in a study among type 2 diabetes-free participants, fasting plasma insulin and insulin resistant measures have been positively correlated with O-phosphoethanolamine downstream product phosphatidylethanolamine [40]. Thus, this finding is consistent with the available evidence in favor of a biological role of sphingolipid metabolism on diabetes.

While the fact that the metabolite subgroups are correlated makes it difficult to separate the relative contribution of the individual subgroups, in BKMR-P analyses, which accommodate highly dimensional correlated variables simultaneously, phenylpropionate, and the medium HDL and small LDL particle concentrations drove most of the observed joint association of metabolites with diabetes. Microbial-related phenylpropionate is positively correlated with whole grain and fruit intake [41], dietary fiber and microbiome diversity [34], and although has been associated to incident type 2 diabetes [34, 35], their precise mechanism of action is unknown. However, it is hypothesized that its antioxidant properties could decrease insulin resistance [34]. Alternatively, type 2 diabetes is frequently preceded by a dyslipidemia characterized by hypertriglyceridemia with low HDL cholesterol levels and reduced LDL size [42], which is induced by an increased hepatic secretion of large VLDL particles that interact with the cholesteryl ester transfer protein and hepatic lipase [42, 43]. The progressive replacement of cholesteryl esters by triglycerides in the HDL particle gradually generates smaller and denser particles [44]. Additional experimental research, however, is needed to further clarify the role of the specific lipoprotein subclasses concentrations on type 2 diabetes development.

The present study is not exempt of limitations. For instance, the fact that only individuals with suggestive evidence of altered non-fasting glucose levels underwent a second measurement in fasting condition allowed for analysis of diabetes status but not for glycaemia as a continuous measure, possibly a more powerful endpoint. Similarly, no information on insulin sensitivity or secretion was available, which could have provided additional insights relative to metabolic pathways in pre-diabetes. Moreover, the limited number of type 2 diabetes cases may not have enough power to detect interactions. The direction of the associations was, however, consistent in all the evaluated subgroups. Additionally, the HLS was derived mainly from self-reported information, thus non-differential miss-classification of the HLS components, which could attenuate the observed associations, cannot be ruled out. Alternatively, differential miss-classification of habits potentially related to social stigma such as alcohol intake may introduce some bias. Nonetheless, self-reported dietary information has been widely used on several other population cohorts. Importantly, the results from our sensitivity analysis support that biases introduced by alcohol are unlikely, although information on binge drinking pattern was unavailable. An additional limitation relates to the fact that metabolomic data was obtained using a targeted approach and only a predefined set of metabolites was available. Thus, we may have missed relevant metabolites. Nonetheless, in our data early lifestyle-related metabolic signatures widely explained the association between a healthy lifestyle and the subsequent occurrence of type 2 diabetes. This assertion is backed by study strengths such as the complex sampling design, which makes our study population representative of the general population from a Spanish region, the prospective study design, and the long follow-up.

## Conclusions

In our population-based sample, we observed a strong, inverse, association of HLS with incident type 2 diabetes, which was substantially explained by differences in lifestyle-related plasma metabolites measured years before the type 2 diabetes clinical diagnosis. Our data support that lifestyle-related metabolic changes have a relevant biological role in type 2 diabetes development, and suggest that metabolomics can contribute to the early identification of individuals who could benefit from intensified lifestyle-related precision interventions for the type 2 diabetes prevention and control.

## Supplementary Information

**Additional file 1. ** Additional findings that further support and/or give further detail to the main results.

## Data Availability

The datasets analyzed during the current study are not publicly available since the steering committee and the participants did not approve unrestricted data sharing. However, data can be accessed upon a reasonable request.

## Abbreviations

HLS: Healthy lifestyle score
BMI: Body mass index
RR: Rate ratio
RD: Rate difference
CI: Confidence interval
HDL: High-density lipoprotein
LDL: Low-density lipoprotein
BKMR-P: Bayesian kernel machine regression-probit
UHRH: University hospital rio hortega
HbA1c: Hemoglobin type A, subfraction 1c (glycated hemoglobin)
aMED: Alternate mediterranean diet score
AHEI: Alternate healthy eating index
MET: Metabolic equivalent of task
NMR: Nuclear magnetic resonance
ppm: parts per million
VLDL: Very low-density lipoprotein
FDR: False discovery rate
PIP: Posterior inclusion probability
IDL: Intermediate-density lipoprotein
MD: Mean difference
EPIC: European prospective investigation into cancer and nutrition cohort
