# Supplementary material for: Healthy lifestyle, metabolomics and incident type 2 diabetes in a population-based cohort from Spain
Source: Int J Behav Nutr Phys Act. 2022 Jan 27;19:8. doi: 10.1186/s12966-021-01219-3 (PMC8793258; doi:10.1186/s12966-021-01219-3)
Supplement: Supplementary file 1 — Additional file 1. Additional findings that further support and/or give further detail to the main results. [file 12966_2021_1219_MOESM1_ESM.pdf]

SUPPLEMENTARY MATERIAL

Supplementary Figure S1. Flow diagram of included participants.

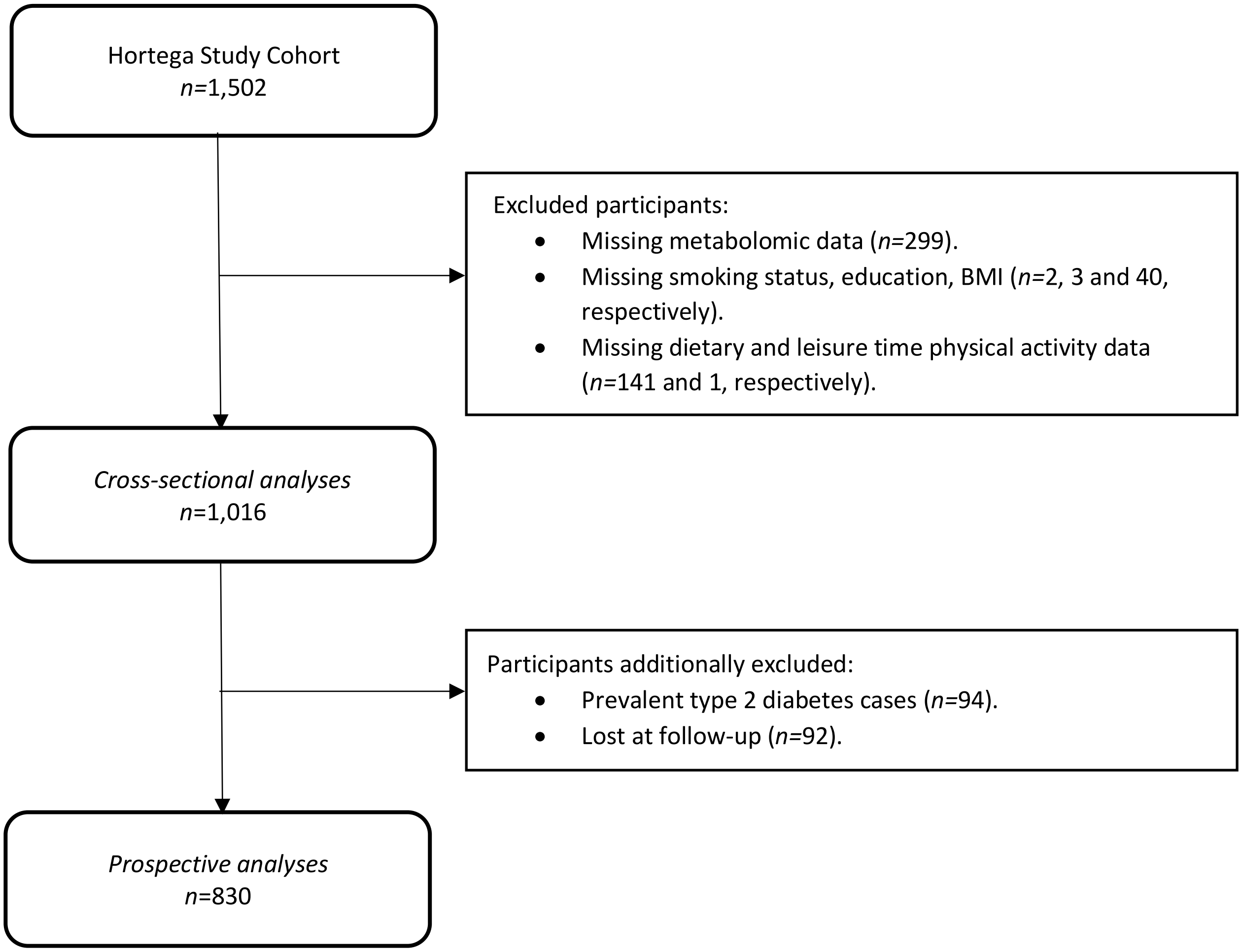

Supplementary Table S1. Differences in participant characteristics by inclusion status into the study population.

Characteristics	Excluded (n=486) [a]	Included (n =1,016)
Age, years, mean	54.2	53.1
Sex, male, %	50.0	50.3
Education, ≤Higher education, %	72.3	71.4
Prevalent Dyslipidaemia, Yes, %	56.8	53.0
Prevalent Hypertension, Yes, %	43.4	43.4

[a] Some of the excluded participants missed education (n=9).

Supplementary Table S2. Adherence to Healthy Lifestyle Score (HLS) components in the Hortega Study (*n* =1,016).

HLS Component	Criteria for 1 point	Adherence, *n* (%)
BMI, kg/m^2	≥18.5 & <25	400 (42.07)
Leisure time physical activity, METs-minute/week	≥600	195 (21.68)
Alcohol consumption, grams/day	Women: ≥5 & ≤15	93 (10.02)
	Men: ≥5 & ≤30	214 (21.51)
8-component aMED*, points	Above the 60th percentile (≥5)	320 (32.10)
Smoking	Never smoker	466 (43.64)

* Alcohol was excluded from the aMED definition.

Supplementary Table S3. Median (IQR) NMR-metabolites levels by HLS categories in Hortega Study participants (*n* =1,016).

			Adherence to HLS		
Group	Metabolite	Overall	Low HLS (0-1 points)	Moderate HLS (2 points)	High HLS (3-5 points)
n (%)		1,016 (100.00)	491 (46.41)	310 (30.39)	215 (23.20)
Lipoprotein profile	*Cholesterol, mmol/L*				
	VLDL cholesterol	0.56 (0.34 - 0.86)	0.67 (0.42 - 0.95)	0.52 (0.33 - 0.86)	0.41 (0.27 - 0.64)
	LDL cholesterol	3.78 (3.17 - 4.49)	3.97 (3.26 - 4.62)	3.84 (3.17 - 4.54)	3.44 (2.92 - 4.12)
	HDL cholesterol	1.73 (1.42 - 2.07)	1.66 (1.36 - 1.96)	1.81 (1.48 - 2.13)	1.78 (1.49 - 2.18)
	IDL cholesterol	0.33 (0.24 - 0.45)	0.37 (0.27 - 0.49)	0.33 (0.23 - 0.43)	0.28 (0.21 - 0.37)
	Triglycerides, mmol/L				
	VLDL triglycerides	1.11 (0.73 - 1.79)	1.31 (0.86 - 2.02)	1.08 (0.69 - 1.78)	0.85 (0.65 - 1.29)
	LDL triglycerides	0.30 (0.25 - 0.36)	0.31 (0.26 - 0.38)	0.30 (0.24 - 0.37)	0.28 (0.24 - 0.32)
	HDL triglycerides	0.19 (0.16 - 0.24)	0.20 (0.16 - 0.25)	0.19 (0.15 - 0.25)	0.18 (0.15 - 0.22)
	IDL triglycerides	0.17 (0.13 - 0.21)	0.18 (0.15 - 0.22)	0.17 (0.13 - 0.21)	0.15 (0.12 - 0.18)
	Lipoprotein particle concentration				
	Large VLDL, *nmol/L*	1.41 (0.90 - 2.48)	1.74 (1.07 - 2.71)	1.37 (0.84 - 2.58)	1.07 (0.73 - 1.82)
	Medium VLDL, *nmol/L*	9.58 (6.78 - 14.51)	10.69 (7.83 - 16.15)	9.15 (6.49 - 14.28)	7.56 (6.13 - 10.59)
	Small VLDL, *nmol/L*	59.90 (38.53 - 98.49)	74.32 (44.58 - 111.25)	58.13 (35.07 - 101.11)	43.83 (32.44 - 69.19)
	Large LDL, *nmol/L*	231.46 (196.97 - 267.76)	237.05 (203.22 - 273.84)	233.68 (200.59 - 261.66)	215.03 (182.98 - 251.08)
	Medium LDL, *nmol/L*	528.73 (438.15 - 653.53)	543.50 (451.43 - 676.81)	524.43 (437.67 - 659.83)	501.34 (407.22 - 604.98)
	Small LDL, *nmol/L*	725.31 (598.56 - 891.49)	773.16 (646.10 - 934.06)	738.21 (601.57 - 885.64)	636.31 (540.18 - 755.05)
	Large HDL, *µmol/L*	0.37 (0.32 - 0.42)	0.36 (0.32 - 0.42)	0.37 (0.32 - 0.44)	0.36 (0.31 - 0.40)
	Medium HDL, *µmol/L*	11.15 (9.25 - 13.35)	10.76 (9.09 - 12.68)	11.60 (9.36 – 14.00)	11.52 (9.32 - 13.61)
	Small HDL, *µmol/L*	23.48 (19.96 - 27.68)	23.16 (19.64 - 27.15)	24.27 (20.36 - 28.56)	23.59 (20.41 - 27.55)
Amino acids*	Alanine	16.44 (15.72 – 17.00)	16.20 (15.49 - 16.85)	16.42 (15.78 - 16.98)	16.85 (16.18 - 17.24)
	Creatine phosphate	1.63 (1.52 - 1.71)	1.61 (1.49 - 1.70)	1.63 (1.53 - 1.70)	1.68 (1.59 - 1.73)

	Creatine	1.84 (1.72 - 1.95)	1.80 (1.68 - 1.92)	1.83 (1.73 - 1.94)	1.91 (1.83 - 1.99)
	Cysteine	1.36 (1.27 - 1.44)	1.34 (1.24 - 1.43)	1.36 (1.27 - 1.43)	1.39 (1.34 - 1.48)
	Glutamine	7.52 (7.03 - 7.96)	7.33 (6.82 - 7.85)	7.49 (7.10 - 7.94)	7.81 (7.42 - 8.07)
	N-acetylglutamine	9.12 (8.71 - 9.42)	9.01 (8.58 - 9.34)	9.09 (8.71 - 9.41)	9.32 (9.03 - 9.54)
	Proline	9.45 (8.92 - 9.93)	9.28 (8.75 - 9.81)	9.45 (9.00 - 9.89)	9.75 (9.38 - 10.11)
	Tryptophan	2.10 (1.91 - 2.29)	2.07 (1.86 - 2.26)	2.10 (1.92 - 2.29)	2.13 (1.97 - 2.34)
	Tyrosine	2.60 (2.42 - 2.75)	2.58 (2.38 - 2.72)	2.59 (2.43 - 2.74)	2.66 (2.53 - 2.80)
	Isoleucine	11.44 (11.02 - 11.72)	11.38 (10.88 - 11.68)	11.44 (10.98 - 11.72)	11.53 (11.20 - 11.81)
	Leucine	9.98 (9.59 - 10.28)	9.87 (9.47 - 10.24)	9.98 (9.58 - 10.27)	10.15 (9.75 - 10.34)
	Valine	10.72 (10.27 - 11.04)	10.66 (10.18 – 11.00)	10.69 (10.24 - 11.03)	10.86 (10.54 - 11.14)
Fatty acids*	CH_2CH_2CO	19.14 (18.28 - 20.59)	19.45 (18.40 - 20.88)	19.23 (18.24 - 20.66)	18.80 (17.87 - 19.68)
	CH_2CH_3	38.54 (37.56 - 39.79)	38.66 (37.69 - 39.99)	38.62 (37.54 - 40.09)	38.21 (37.33 – 39.00)
	CH_2N	145.29 (133.27 - 163.49)	151.26 (136.11 - 170.56)	146.54 (134.24 - 163.80)	139.37 (129.74 - 148.93)
	CH_3	80.16 (77.83 - 82.36)	80.64 (78.06 - 82.85)	80.12 (78.02 - 82.14)	79.51 (77.27 - 81.62)
	$CHCH_2CH$	12.52 (11.99 - 13.17)	12.57 (11.99 - 13.22)	12.65 (11.99 - 13.27)	12.28 (11.94 - 12.86)
	Isobutyrate	6.84 (6.48 - 7.08)	6.77 (6.39 - 7.04)	6.81 (6.45 - 7.08)	6.97 (6.67 - 7.15)
Fluid balance*	Albumin	9.43 (8.85 - 9.99)	9.26 (8.59 - 9.87)	9.36 (8.86 – 10.00)	9.72 (9.32 - 10.20)
	Creatinine	3.19 (3.05 - 3.29)	3.16 (3.02 - 3.27)	3.19 (3.05 - 3.29)	3.23 (3.09 - 3.33)
Energy metabolism*	*Glycolysis*				
	Citrate	4.43 (4.10 - 4.71)	4.33 (3.98 - 4.63)	4.42 (4.10 - 4.68)	4.59 (4.34 - 4.81)
	Lactate	21.50 (19.46 - 24.76)	22.30 (19.77 - 25.16)	21.48 (19.34 - 24.88)	20.68 (18.77 - 23.23)
	Pyruvate	1.80 (1.70 - 1.91)	1.77 (1.66 - 1.88)	1.80 (1.71 - 1.89)	1.87 (1.79 - 1.96)
	Ketone bodies				
	Acetate	5.49 (5.22 - 5.71)	5.43 (5.11 - 5.64)	5.46 (5.24 - 5.71)	5.62 (5.42 - 5.77)
	Acetone	6.75 (6.20 - 7.63)	6.91 (6.36 - 7.82)	6.81 (6.15 - 7.63)	6.48 (6.04 - 7.06)
	3-Hydroxybutyrate	7.92 (7.40 - 8.35)	7.74 (7.19 - 8.25)	7.88 (7.44 - 8.31)	8.14 (7.85 - 8.50)
Products of bacterial co-metabolism*	Ethanol	8.54 (7.93 - 9.12)	8.45 (7.86 - 9.08)	8.52 (7.92 - 9.10)	8.66 (8.01 - 9.13)
	Isopropanol	6.46 (6.09 - 6.80)	6.36 (5.95 - 6.72)	6.46 (6.13 - 6.79)	6.65 (6.34 - 6.91)

	Methanol	0.71 (0.65 - 0.78)	0.70 (0.64 - 0.77)	0.72 (0.66 - 0.78)	0.72 (0.67 - 0.80)
	Trimethylamines	6.32 (5.86 - 6.71)	6.18 (5.71 - 6.61)	6.26 (5.86 - 6.71)	6.52 (6.22 - 6.87)
	Phenylpropionate	13.41 (12.90 - 13.90)	13.20 (12.60 - 13.80)	13.40 (12.90 - 13.90)	13.70 (13.30 - 14.00)
Phosphoethanolamines*	O-phosphoethanolamine	6.01 (5.62 - 6.32)	5.92 (5.51 - 6.26)	5.99 (5.64 - 6.31)	6.18 (5.90 - 6.40)

Abbreviations: IQR, interquartile range; NMR, nuclear magnetic resonance; HLS, Healthy lifestyle score.

* We normalized the spectral vector to the total spectral area excluding residual water signals to minimize the effects of variable dilution of the sample. The metabolic content is therefore expressed in relative metabolic content (unitless).

Supplementary Table S4. Participants' characteristics by incident type 2 diabetes in Hortega Study participants without prevalent type 2 diabetes at baseline (*n*=830).

Characteristics	Overall	Incident type 2 diabetes	Non-incident type 2 diabetes
n (%)	830 (100.0)	51 (5.11)	779 (94.89)
Age, years, mean (SE)	47.89 (16.52)	60.36 (14.98)	47.21 (16.33)
Sex, male, *n* (%)	400 (46.65)	22 (39.09)	378 (47.06)
Education, ≤Higher education, *n* (%)	597 (69.95)	48 (92.76)	549 (68.72)
Energy intake, Kilocalories/day, mean (SE)	2,206.17 (709.22)	1,999.10 (731.53)	2,217.33 (706.29)
Prevalent Dyslipidaemia, Yes, *n* (%)	435 (51.69)	35 (69.89)	400 (50.71)
Prevalent Hypertension, Yes, *n* (%)	334 (34.38)	45 (86.17)	289 (31.59)
Healthy Lifestyle Score (HLS) components			
BMI, kg/m^2, mean (SE)	26.07 (4.07)	31.58 (4.75)	25.78 (3.81)
Leisure time physical activity, METs-minute/week, mean (SE)	477.18 (1,351.88)	71.78 (211.24)	499.03 (1,383.60)
8-point aMED (alcohol excluded), mean (SE)	3.73 (1.63)	3.98 (1.55)	3.72 (1.63)
Alcohol intake, grams/day, mean (SE)	11.49 (20.66)	8.31 (12.74)	11.66 (20.99)
Smoking status, Never smoker, *n* (%)	380 (43.75)	28 (50.40)	352 (43.39)

Abbreviations: aMED, alternate Mediterranean Diet score; BMI, body mass index; MET, Metabolic Equivalent of Task; SE, standard error.

Supplementary Table S5. Rate Ratio (95% Confidence interval) for type 2 diabetes incidence per 1-point HLS increase in prevalent type 2 diabetes-free Hortega Study participants (*n=830*).

HLS categories (points)	Low (0-1)	Medium (2)	High (3-5)	Per 1-point increase	p-linear trend
Cases/Non-cases	32/361	17/243	2/175	51/779	
Model 1, RR (95% CI)	1.00 (Reference)	0.83 (0.44 - 1.56)	0.19 (0.04 - 0.83)	0.68 (0.50 - 0.91)	0.009
Model 2, RR (95% CI)	1.00 (Reference)	0.83 (0.44 – 1.56)	0.20 (0.04 – 0.88)	0.69 (0.51 - 0.93)	0.01

Abbreviations: CI, confidence interval; HLS, Healthy lifestyle score; RR, rate ratio.

Model 1 was adjusted for age (years), sex (men, women), education (≤ high school, > high school).
Model 2 was model 1 further adjusted for prevalent hypertension (no, yes), total plasma cholesterol (mg/dL) and use of lipid lowering medication (no, yes).

Supplementary Table S6. Rate difference (95% Confidence interval) (per 10,000 person-years) for type 2 diabetes incidence per 1-point HLS increase in prevalent type 2 diabetes-free Hortega Study participants (*n=830*).

HLS categories (points)	Low (0-1)	Medium (2)	High (3-5)	Per 1-point increase	p-linear trend
Cases/Non-cases	32/361	17/243	2/175	51/779	
Model 1, RD (95% CI)	1.00 (Reference)	-10.26 (-37.42, 16.89)	-27.90 (-47.57, -8.24)	-9.86 (-17.92, -1.80)	0.02
Model 2, RD (95% CI)	1.00 (Reference)	-7.15 (-34.31, 20.01)	-24.47(-43.87, -5.06)	-8.23 (-16.34, -0.13)	0.046

Abbreviations: CI, confidence interval; HLS, Healthy lifestyle score; RD, rate difference.

Model 1 was adjusted for age (years), sex (men, women), education (≤ high school, > high school).
Model 2 was model 1 further adjusted for prevalent hypertension (no, yes), total plasma cholesterol (mg/dL) and use of lipid lowering medication (no, yes).

Supplementary Table S7. Rate Ratio and Rate differences/10,000 person-years (95% Confidence interval) for incident type 2 diabetes per 1-point HLS increase when alcohol is included or excluded from aMED and HLS scores in Hortega Study participants without type 2 diabetes mellitus at baseline (n=830).

	Alcohol excluded from aMED only*	Alcohol included in aMED & HLS*	Alcohol excluded from aMED & HLS†	1 point in HLS for non-drinkers*
RR (95%CI) ‡	0.69 (0.51 - 0.93)	0.69 (0.52 - 0.91)	0.59 (0.42 - 0.84)	0.75 (0.57 – 0.99)
p-value	0.01	0.01	0.003	0.045
RD (95%CI) §	-8.23 (-16.32, -0.14)	-8.00 (-15.41, -0.59)	-12.91 (-22.75, -3.07)	-6.17 (-14.79, 2.45)
p-value	0.047	0.03	0.01	0.16

Abbreviations: aMED, Alternate Mediterranean Diet; HLS, Healthy Lifestyle Score; RD, rate difference; RR, rate ratio; CI, confidence interval.

* Model adjusted for age (years), sex (men, women), education (≤ high school, > high school), prevalent hypertension (no, yes), total plasma cholesterol (mg/dL) and use of lipid lowering medication (no, yes).

† Model adjusted for age (years), sex (men, women), education (≤ high school, > high school), prevalent hypertension (no, yes), total plasma cholesterol (mg/dL) and use of lipid lowering medication (no, yes), and alcohol intake (g/day).

‡ Rate ratios obtained from Poisson regression with the individual follow-up time in log person-years modelled as an offset term.

§ Rate differences obtained from Aalen additive hazard regression models.

Supplementary Table S8. Rate ratio (RR) (95% CI) for incident Type 2 Diabetes Mellitus per 1-point HLS increase by subgroups in Hortega Study participants without baseline type 2 diabetes mellitus (*n=830*).

Subgroup	Cases / Non cases	RR (95% CI) *	p-interaction
Overall	51/779	0.69 (0.51 - 0.93)	
Sex			
Female	29/401	0.67 (0.46 - 0.97)	0.78
Male	22/378	0.73 (0.46 - 1.16)	
Education			
≤High school	48/549	0.68 (0.50 - 0.93)	0.71
>High school	3/230	0.77 (0.42 - 1.42)	
Prevalent dyslipidaemia			
No	16/379	0.66 (0.40 - 1.09)	0.79
Yes	35/400	0.71 (0.53 - 0.95)	
Prevalent hypertension			
No	6/490	0.42 (0.19 - 0.95)	0.18
Yes	45/289	0.76 (0.55 - 1.04)	

Abbreviations: CI, confidence interval; HLS, Healthy lifestyle score; RR, rate ratio.

*Poisson regression model adjusted for age (years), sex (men, women), education (≤ high school, > high school), prevalent hypertension (no, yes), total plasma cholesterol (mg/dL) and lipid lowering medication (no, yes). The individual follow-up in log person-years was modelled as an offset term.

Supplementary Figure S2. Overall association of the metabolites mixture with type 2 diabetes incidence (difference in the probit of incident diabetes hazard and 95% credibility intervals) when all predictors are at a particular percentile compared with the value when all of them are at their 50th percentile. BKMR-P models adjusted for age (years), sex (men, women), education (≤high school, >high school), total cholesterol level (mg/dl), lipid-lowering medication (yes/no) and hypertension (yes/no).

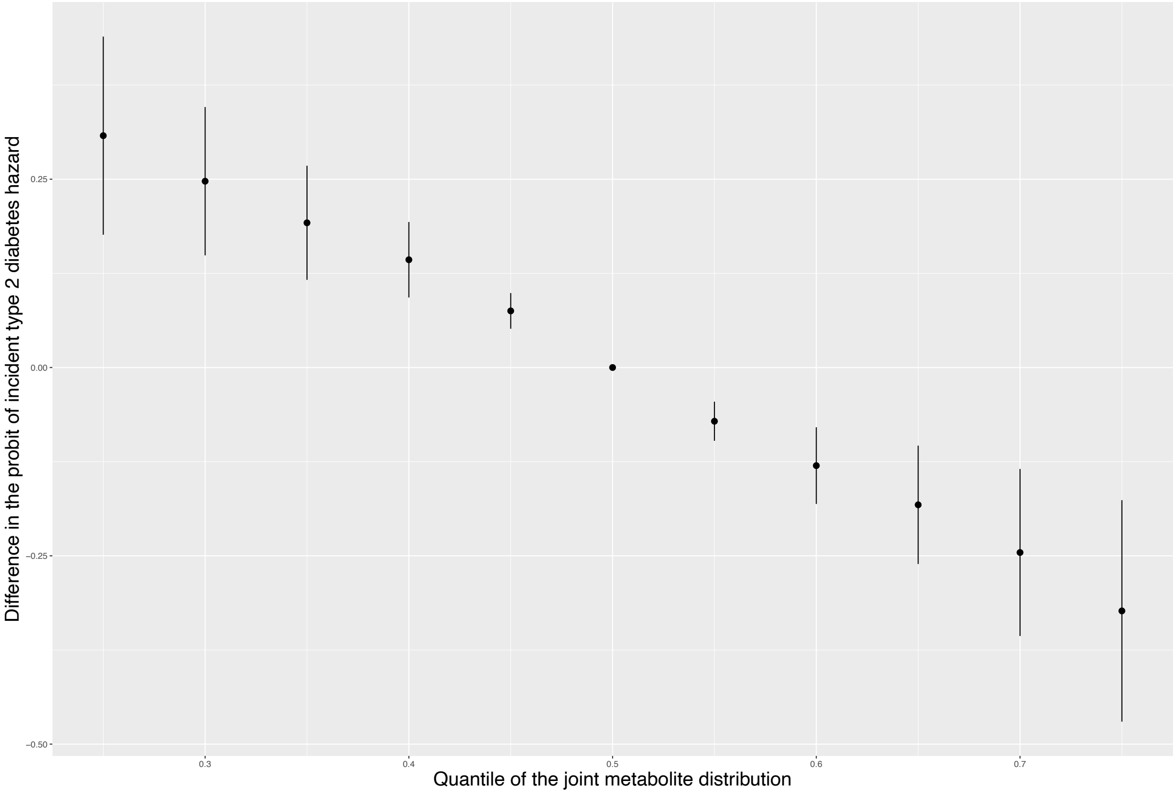

Supplementary Figure S3. Single-metabolite dose-response association of selected metabolites a posterior inclusion probabilities (PIP) greater than 20% with incident type 2 diabetes when all the other metabolites are fixed to their 50th percentile, estimated from BKMR-P models adjusted for age (years), sex (men, women), education (≤high school, >high school), total cholesterol level (mg/dl), lipid-lowering medication (yes/no) and hypertension (yes/no). h(*z*) -blue line- is the difference in the probit of incident type 2 diabetes hazard. Grey shades indicate the 95% credibility intervals.

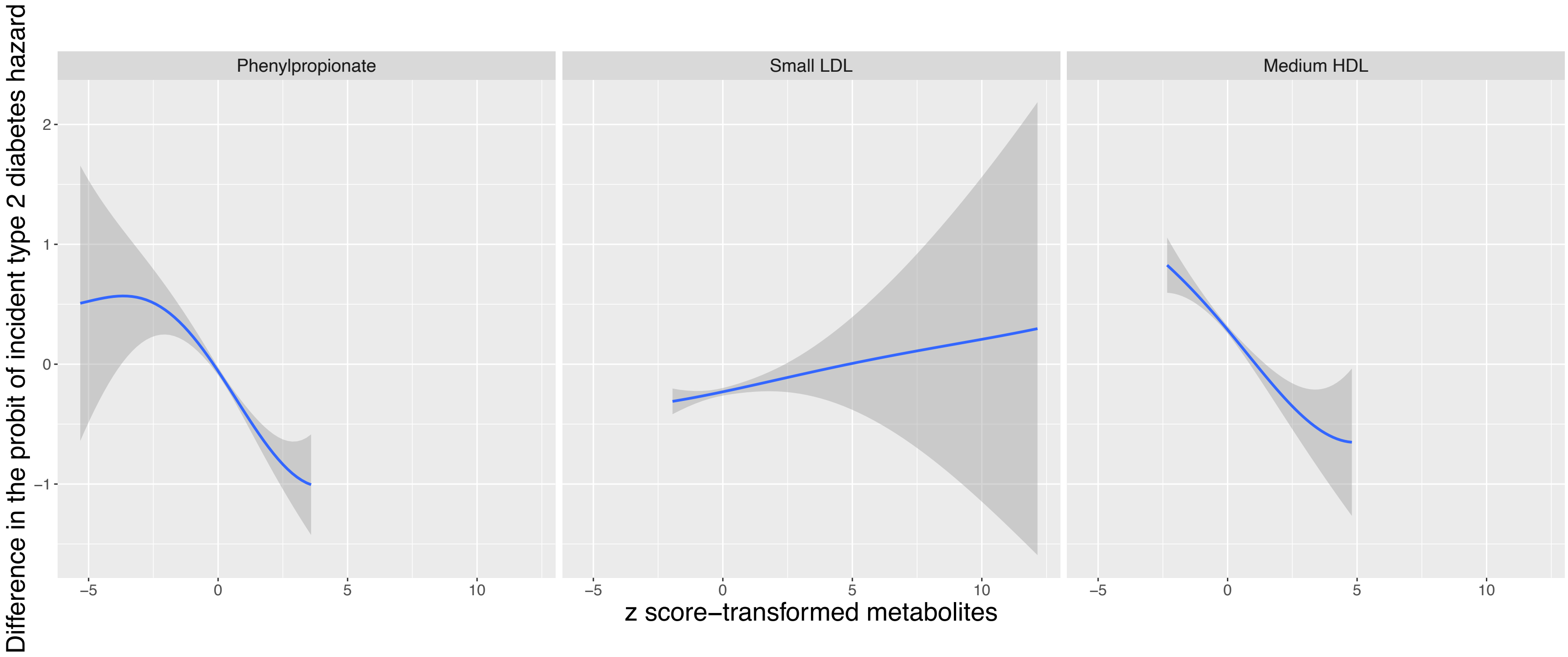

Supplementary Table S9. Posterior Inclusion Probabilities (PIP) of individual lifestyle-related plasma metabolites in the BKMR-P model.

Group	Metabolites	PIP
Amino acids	Alanine	0
	Creatine phosphate	0.06
	Creatine	0
	Cysteine	0.02
	Glutamine	0
	Proline	0.05
	Tryptophan	0
	Tyrosine	0
	Isoleucine	0
	Leucine	0
	Valine	0
	N-acetylglutamine	0
Fatty acids	CH_2CH_2CO	0
	CH_2N	0.08
	Isobutyrate	0
Fluid balance	Albumin	0
	Creatinine	0
Energy metabolism	Acetate	0
	Acetone	0
	3-Hydroxybutyrate	0.02
	Citrate	0
	Pyruvate	0
Products of bacterial co-metabolism	Isopropanol	0
	Trimethylamines	0
	Phenylpropionate	1
Phosphoethanolamines	O-phosphoethanolamine	0
Lipoparticles concentrations and particle subclasses	VLDL cholesterol	0
	LDL cholesterol	0
	HDL cholesterol	0.03
	IDL cholesterol	0
	VLDL triglycerides	0
	IDL triglycerides	0
	Large VLDL	0
	Medium VLDL	0
	Small VLDL	0
	Small LDL	0.23
	Medium HDL	1

BKMR-P models adjusted for age (years), sex (men, women), education (≤high school, >high school), total cholesterol level (mg/dl), lipid-lowering medication (yes/no) and hypertension (yes/no).

Supplementary Table S10. Avoided type 2 diabetes incidence cases per 10,000 person-years per 1-point HLS increase not attributable (direct effect) and attributable (mediated effect) to changes in specific metabolites using the 'product of coefficients method' after accounting for other relevant metabolites within the group.

	Fully adjusted mediator model further adjusted for the other metabolites within the group	Outcome model without the specific metabolite after adjustment for the other metabolites within the group	Outcome model with the specific metabolite after adjustment for the other metabolites within the group			
				Direct effect (95% CI)	Mediated effect [b]	
Fully adjusted models further adjusted for other relevant metabolites from BKMR analysis[c]	Difference in metabolite by a 1-point increase in the HLS (95% CI)	Absolute change in diabetes cases per 1-point HLS increase, per 10000 person-years (95% CI) [a]	Absolute change in diabetes cases per 1-unit increase in metabolite, per 10000 person-years (95% CI) [a]	Absolute change in diabetes cases per 1-point HLS increase, per 10000 person-years (95% CI) [a]	Difference in change, per 10000 person-years, (95 % CI)	Percentage of adjusted change, %
Phenylpropionate, unitless	0.075 (0.0281, 0.1219)	-6.41 (-15.57, 2.77)	-31.43 (-53.38, -9.46)	-4.09 (-13.49, 5.29)	-2.36 (-4.97, -0.48)	36.8
Medium HDL, *µmol/L*	0.1441 (-0.0162, 0.3045)	-4.69 (-14.28, 4.92)	-5.03 (-11.49, 1.42)	-4.09 (-13.50, 5.29)	-0.73 (-2.36, 0.27)	15.5
Small LDL, *nmol/L*	-1.9442 (-10.3698, 6.4814)	-4.31 (-13.6, 5.00)	0.06 (-0.08, 0.20)	-4.09 (-13.50, 5.29)	-0.11 (-1.14, 0.66)	2.7

Abbreviations: CI, confidence interval.

Models adjusted for age (years), sex (men, women), education (≤ high school, > high school), prevalent hypertension (no, yes), total plasma cholesterol (mg/dL) and use of lipid lowering medication (no, yes).

[a]Absolute changes in diabetes incidence (per 10,000 person-years) associated with one-unit increase in HLS and in metabolites were obtained from Aalen additive hazards models.

[b]Effects mediated by metabolites were estimated with the 'product of coefficients method' that multiplies the coefficient for the mean change in metabolites for a one-unit change in HLS from the mediator model (first column of this table) by the absolute change in diabetes incidence cases associated with one-unit increase in metabolite (third column of this table) expressed in absolute terms (difference in change reflecting the number of avoided diabetes cases per 10000 person-years) and relative to the adjusted changes in diabetes cases before adding the metabolites to the model (second column of this table). The 95% confidence intervals (CIs) in the table were derived by simulation from the estimated model coefficients and covariance matrices.

[c]Relevant metabolites defined as showing a PIP > 20 % in BKMR-P regression.
